# Electrical and Optical Properties of Silicon Oxide Lignin Polylactide (SiO_2_-L-PLA)

**DOI:** 10.3390/molecules25061354

**Published:** 2020-03-16

**Authors:** Jacek Fal, Katarzyna Bulanda, Julian Traciak, Jolanta Sobczak, Rafał Kuzioła, Katarzyna Maria Grąz, Grzegorz Budzik, Mariusz Oleksy, Gaweł Żyła

**Affiliations:** 1 Department of Experimental Physics, Faculty of Mathematics and Applied Physics, Rzeszów University of Technology, 35-959 Rzeszów, Poland; jacekfal@prz.edu.pl (J.F.); jtraciak@prz.edu.pl (J.T.); jolantasobczak3@gmail.com (J.S.); 2 Department of Polymer Composites, Faculty of Chemistry, Rzeszów University of Technology, 35-959 Rzeszów, Poland; katarzyna.bulanda@o2.pl (K.B.); molek@prz.edu.pl (M.O.); 3 Department of Surface Engineering, Institute of Materials Engineering, Faculty of Engineering and Technical Sciences in Stalowa Wola, The John Paul II Catholic University of Lublin, 37-450 Stalowa Wola, Poland; rafalkuziola@kul.pl (R.K.); katarzyna.graz@kul.pl (K.M.G.); 4 Department of Mechanical Engineering, Faculty of Mechanical Engineering and Aeronautics, Rzeszów University of Technology, 35-959 Rzeszów, Poland; gbudzik@prz.edu.pl

**Keywords:** lignin, silicon oxide, polylactide, electrical properties, optical properties

## Abstract

This paper presents a study on the electrical properties of new polylactide-based nanocomposites with the addition of silicon-dioxide–lignin nanoparticles and glycerine as a plasticizer. Four samples were prepared with nanoparticle mass fractions ranging between 0.01 to 0.15 (0.01, 0.05, 0.10, and 0.15), and three samples were prepared without nanoparticle filler—unfilled and unprocessed polylactide, unfilled and processed polylactide, and polylactide with Fusabond and glycerine. All samples were manufactured using the melt mixing extrusion technique and injection molding. Only the unfilled and unprocessed PLA sample was directly prepared by injection molding. Dielectric properties were studied with broadband spectroscopy in a frequency range from 0.1 Hz to 1 MHz in 55 steps designed on a logarithmic scale and a temperature range from 293.15 to 333.15 K with a 5 K step. Optical properties of nanocomposites were measured with UV-VIS spectroscopy at wavelengths from 190 to 1100 nm. The experimental data show that the addition of silicon-dioxide–lignin and glycerine significantly affected the electrical properties of the studied nanocomposites based on polylactide. Permittivity and electrical conductivity show a significant increase with an increasing concentration of nanoparticle filler. The optical properties are also affected by nanofiller and cause an increase in absorbance as the number of silicon-dioxide–lignin nanoparticles increase.

## 1. Introduction

Plastics, due to their properties (e.g., strength, water resistance, thermal and chemical resistance), are popular in almost all areas of our lives, from food packaging to high-end industries. The malleability of plastic allows for objects with sophisticated shapes to be prepared. Plastics are known for their chemical resistance and insulating properties. One of the major reasons for the popularity of plastics is the ratio of the density to the mechanical strength, which is favorable when plastics need to reduce mass and maintain good mechanical properties. Optical properties of plastics are also important. These materials can be transparent or dyed with different colors without leading to changes in other properties.

Despite such advantages, plastics also have a few undesirable features. The most important of which are arguably that many plastics can be environmentally toxic. Therefore, it is important to undertake research on the use of biodegradable materials such as polylactide (PLA) or polyethylene terephthalate (PET).

Market analysis from recent years shows that 50% of the worldwide production capacity of thermoplastic bio-plastics is made up of unmodified starch, while the rest of the market is divided between polycaprolactone (PCL), poly-3-hydroxybutyrate (PHB), poly(3-hydroxybutyrate co-3-hydroxywalate) (PHBV), and poly(lactic acid) (PLA) [1,2].

Poly(lactic acid) is an aliphatic polymer obtained from, among others, corn, sugar, and sugar cane, by the fermentation of sugar [3]. PLA was introduced to the market as an alternative to fossil-fuel-based polymers [4,5]. It has good mechanical properties, but as a material, it is relatively expensive, and some properties—such as brittleness and low heat resistance—limit its use [6,7]. PLA is completely biodegradable. It decomposes by hydrolysis to lactic acid, which is converted by microbes into carbon monoxide and water [8]. Composting PLA together with other types of biomass leads to biodegradation in two weeks, and the material completely degrades in 3–4 weeks [9]. PLA is suitable in applications requiring interaction with the human body without causing harmful effects to health. Additionally, it is safe for food and medical use [10,11]. PLA is used in orthopedics when high mechanical strength and ductility are required [12]. Some researchers have proposed combining PLA with filler to improve the properties of PLA and remove some of its disadvantages [13,14,15].

Lignin is one of the most abundant renewable bio-resources that can be used for this purpose. Chemically speaking, lignin is an amorphous polyphenolic macromolecule that consists of a large number of polar functional groups [13,16]. These phenolic groups are -OH, carbonyl, alkyl aryl ether, biophenyl, aliphatic hydroxyl, diaryl ehter, phenylopropane, guaiacyl, etc. [17,18]. Details on the functionality of these groups are described in the literature [17,18]. Lignin provides mechanical support for the plant and ensures rigidity, the internal transport of water, nutrients, and protection against microorganism attacks [19,20].

In the paper industry, pulping is performed to eliminate lignin and hemicellulose from wood to make cellulose pulp [21]. It is estimated that, worldwide, approximately 70 million tons of lignin is produced every year as a by-product of the pulp and paper industrial processes [21]. Only about 2% of total lignin produced is used as concrete additives, stabilizers or dispersants, and surfactants. The rest of the production is treated as a heating fuel or as waste [22,23].

Lignin can be used as a value-added component in various fields, including chemicals, fuels, carbon fibers, pharmaceutical products, and electrical materials [24,25,26,27]. The biodegradability of lignin makes it promising as a filler in the field of organic fillers or as a chemical component in polymer blends. Lignin reduces the cost of the end product and affords thermoplastic polymers certain biodegradable properties [28]. Moreover, lignin exhibits antioxidant and antimicrobial properties [29,30,31].

Many researchers have tried to remedy the weaknesses of PLA by combining it with other materials of biological origin. According to some studies, the addition of lignin makes the composite more resistant to heat and moisture [13,14]. Moreover, lignin serves as a stabilizer, preventing the oxidation of plastic composites [32]. Lignin is also considered as a strengthening agent that can improve the thermal and optical properties of PLA. However, the simple mixing of lignin and PLA has reduced the mechanical strength of PLA because of its weak dispersion and interfacial interactions [13,33]. It was found that lignin is not compatible with the majority of aliphatic polyesters (PLA, PBS, and PCL), thus deteriorating the mechanical properties of these composites [16,34]. It has been shown that this deterioration, which is a consequence of the lignin addition, can be overcome by adding coupling agents [35,36].

This paper reports on the preparation of SiO${}_{2}$L-PLA nanocomposites with various mass fractions of filler and their optical and electrical properties studied as a function of temperature and frequency. Our findings show significant impact of silicon oxide–lignin filler on electrical and optical properties of polylactide based nanocomposites.

## 2. Results and Discussion

### 2.1. SEM Investigation

**Figure 1:**
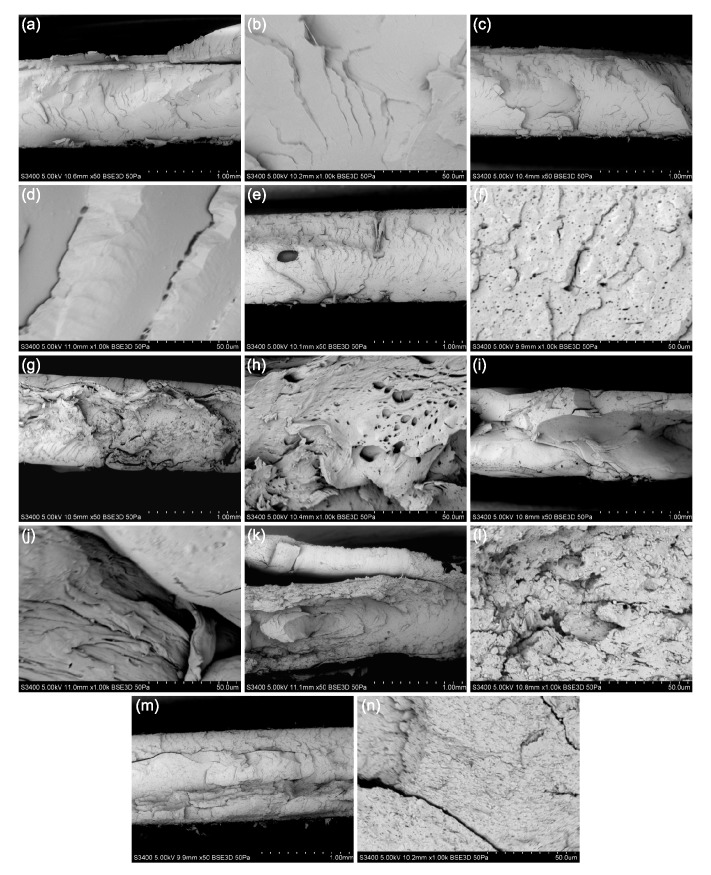
SEM images of fractured surface morphology for (**a**) uuPLA, magnification 50× *g*, (**b**) uuPLA, magnification 1000×, (**c**) upPLA, magnification 50× *g*, (**d**) upPLA, magnification 1000× *g*, (**e**) PLA-FG, magnification 50× *g*, (**f**) PLA-FG, magnification 1000× *g*, (**g**) SiO${}_{2}$L-PLA-FG-1, magnification 50× *g*, (*h*) SiO${}_{2}$L-PLA-FG-1, magnification 1000× *g*, (**i**) SiO${}_{2}$L-PLA-FG-5, magnification 50× *g*, (**j**) SiO${}_{2}$L-PLA-FG-5, magnification 1000× *g*, (**k**) SiO${}_{2}$L-PLA-FG-10, magnification 50× *g*, (**l**) SiO${}_{2}$L-PLA-FG-10, magnification 1000× *g*, (**m**) SiO${}_{2}$L-PLA-FG-15, magnification 50× *g*, and (**n**) SiO${}_{2}$L-PLA-FG-15, magnification 1000× *g*.

Figure 1 shows the scanning electron microscopy of the fractured surface of SiO${}_{2}$-PLA-FG nanocomposites and three composites without any particles. The observed surfaces show the effect of the addition of the plasticizer and the particles. Fractures of both unprocessed (uuPLA—Figure 1a,b) and processed (upPLA—Figure 1c,d) polylactide without glycerine are brittle, and the surface is smooth, which is often observed with brittle fractures, as also shown by others [9,37]. The PLA-FG (Figure 1e,f) composite also shows signs of brittle fracture, but the surface is more jagged. Additionally, many small air bubbles were visible, and these are related to the presence of the compatibilizer and glycerine. SEM images for SiO${}_{2}$L-PLA-FG nanocomposites are presented in Figure 1g–n. There are no visible agglomerates of silicon-dioxide–lignin nanoparticles, which indicates the effectiveness of the dispersion of filler. The fractured surfaces of SiO${}_{2}$L-PLA-FG nanocomposites are characterized by a fibrous and rough appearance, which are the effects of lignin and silicon dioxide, respectively.

### 2.2. Visual Appearance and Absorbance

**Figure 2:**
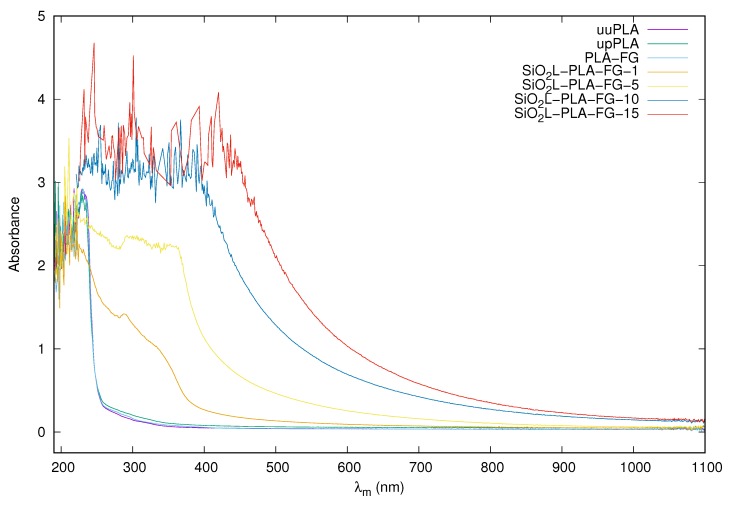
Absorbance spectra for SiO${}_{2}$L-PLA-FG nanocomposites.

**Figure 3:**
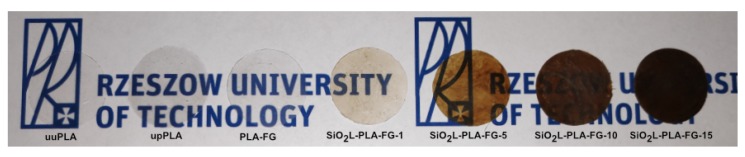
Visual apperance of SiO${}_{2}$-PLA-FG nanocomposites. PLA–polylactide, F–fusabond, G–glycerin.

The UV-VIS spectra for the silicon-dioxide–lignin polylactide nanocomposites with various mass fractions of filler and for three unfilled polymers are presented in Figure 2. The molecules of the compounds tested have chromophore moieties that are responsible for the color. Within them, electronic transitions occur; i.e., the electrons are moved from the ground state to the excited state. The first three samples—uuPLA, upPLA, and PLA-FG—absorb only in the UV range (190–240 nm). As the addition of SiO${}_{2}$L particles increases, the bands are widened and shifted to the right, towards larger wavelengths and towards the VIS, the so-called batochromic move (redshift). As the additive mass fraction increases, the absorbance increases. Increasing absorbance with an increasing mass fraction of silicon-dioxide–lignin particles can be confirmed by digital photography, which is presented in Figure 3 The visual appearance of the unfilled samples confirms a very similar transparency. Comparable results were presented by [38] for cellulose polylactide nanocomposites. Samples with a low particle content also show some transparency; however, in comparison to the unfilled PLA, they are clearly smaller, and decrease with an increasing load of SiO${}_{2}$L filler, becoming practically opaque at the highest tested mass fraction (0.15).

### 2.3. Electrical Properties

**Figure 4:**
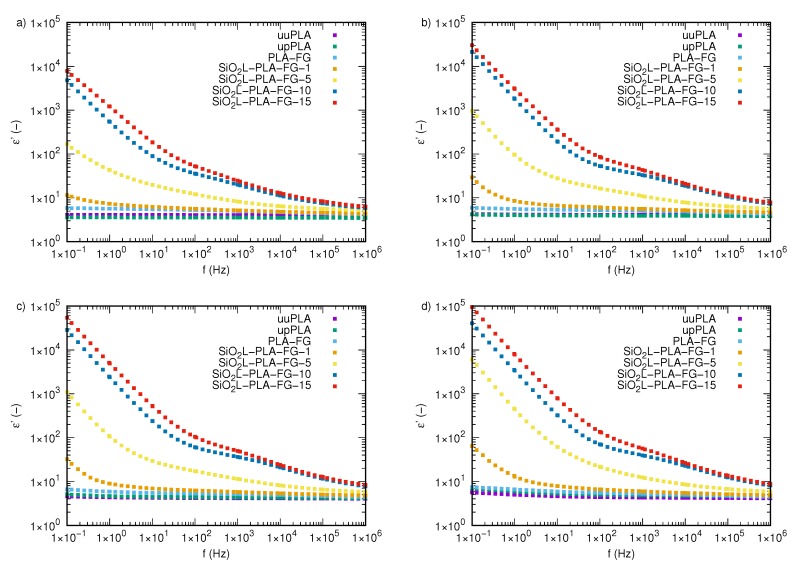
The real part of the complex permittivity of silicon-dioxide–lignin nanocomposites as a function of frequency for (**a**) 293.15 K, (**b**) 323.15 K, (**c**) 328.15 K, and (**d**) 333.15 K.

**Figure A1:**
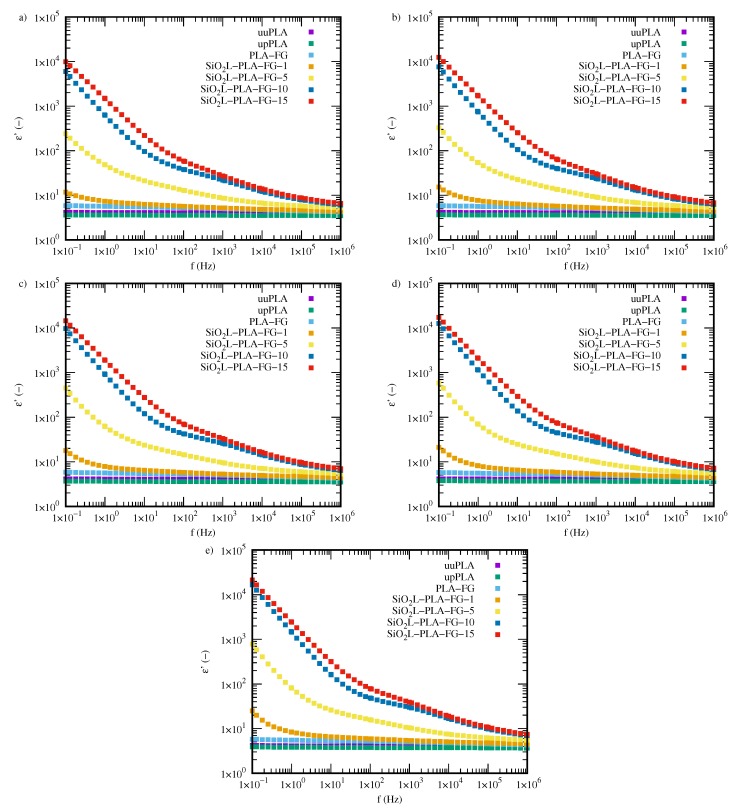
The real part of complex permittivity of silicon-dioxide–lignin nanocomposites as a function of frequency for (**a**) 298.15 K, (**b**) 303.15 K, (**c**) 308.15 K, (**d**) 313.15 K, and (**e**) 318.15 K.

**Figure A2:**
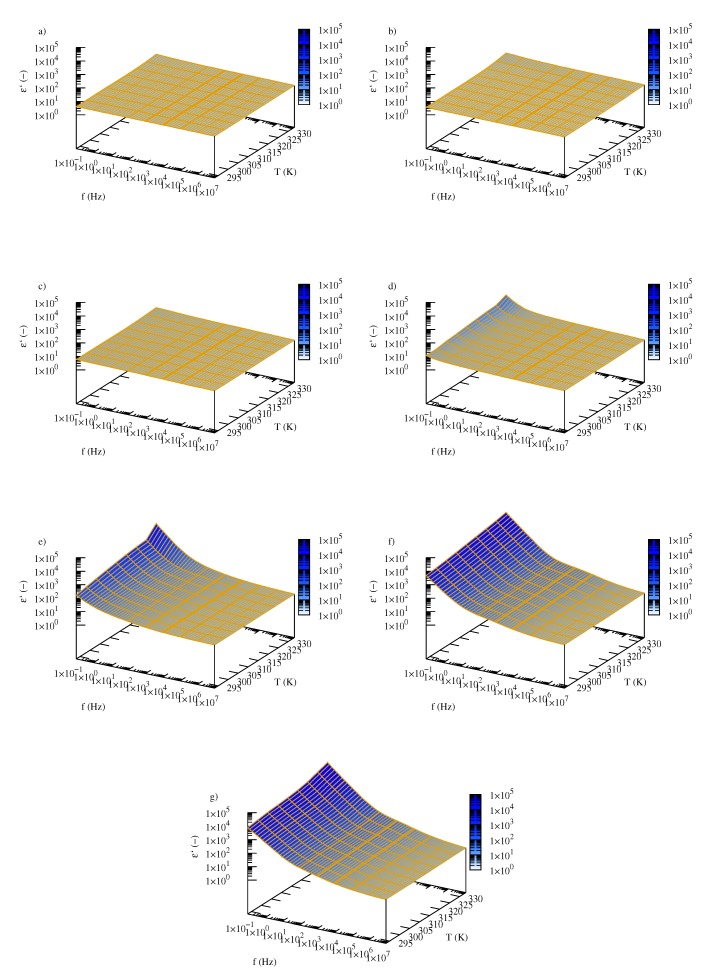
The real part of the permittivity, $\varepsilon {}^{\prime }$, of silicon-dioxide–lignin polylactide nanocomposites as a function of frequency and temperature for (**a**) unprocessed and unfilled PLA, (**b**) unfilled PLA, (**c**) PLA with FB and GL, (**d**) 0.01 mass fraction, (**e**) 0.05 mass fraction, (**f**) 0.10 mass fraction, and (**g**) 0.15 mass fraction.

The dependence of the real part of the complex permittivity, $\varepsilon {}^{\prime },$ on the frequency, *f*, of nanocomposites with various mass fractions, $\varphi_{m}$, of SiO${}_{2}$L filler at four chosen temperatures (293.15, 323.15, 328.15, and 333.15 K) is presented in Figure 4a–d, respectively. The dependence of the real part of the permittivity on the frequency at other examined temperatures is presented in Figure A1. The dielectric properties of the samples without the addition of silicon-dioxide–lignin nanoparticles are almost unaffected by frequency changes, and the observed temperature effect is very weak. Nanocomposites with filler show strong variations in permittivity values, particularly at low frequencies, where decreasing permittivity with increasing frequency can be observed for all tested temperatures and mass fractions of silicon-dioxide–lignin nanoparticles. For higher frequencies, both temperature and frequency effects are much weaker. The frequency dependence of the real part of the complex permittivity, on *f* for samples with different concentrations of silicon-dioxide–lignin nanoparticles and for the three samples without nanoparticles is distinct at each tested temperature. The dielectric spectra of all unfilled samples are mostly unaffected by frequency and, therefore, remain almost constant at all tested frequencies, which is shown in Figure A2a–c. Furthermore, an increase in temperature does not cause significant changes in the values of the real part of the permittivity, excluding the highest tested temperature, where, below 10 Hz, a slight increase in $\varepsilon {}^{\prime }$ with decreasing frequency is visible (Figure 4d). Of note, is that polylactide, after being processed (upPLA) in an extruder, shows a slightly lower permittivity than unprocessed PLA (uuPLA) at temperatures between 293.15 and 323.15 K (Figure 4 and Figure A1) for frequencies below 10 kHz. Above this, the difference is not clear. At 328.15 K (Figure 4c) and 333.15 K (Figure 4d), processed PLA shows a moderately higher permittivity than unprocessed PLA.

**Figure 5:**
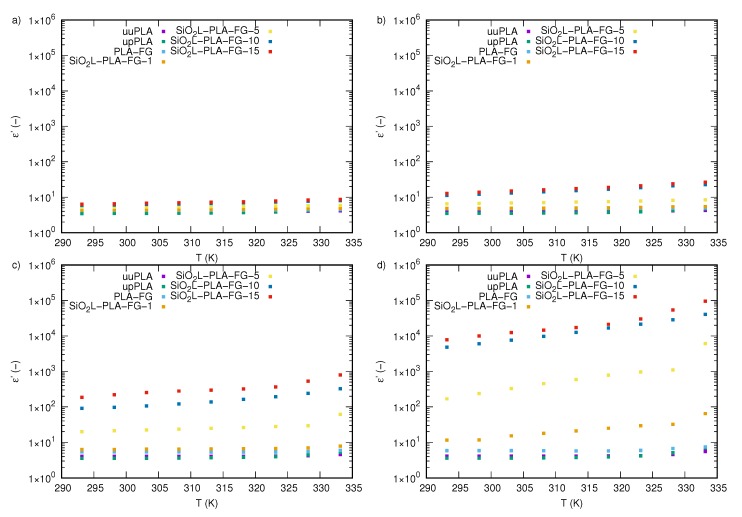
The real part of the complex permittivity of silicon-dioxide–lignin nanocomposites as a function of temperature for (**a**) 0.1 Hz, (**b**) 10 Hz (**c**) 1 kHz, and (**d**) 1 MHz.

The filler (SiO${}_{2}$L) effect is readily seen in all samples with nanoparticles. An increase in the mass fraction of SiO${}_{2}$L nanoparticles causes a significant increase in the real part of the permittivity of the nanocomposite. The effect increases with an increasing mass fraction of silicon-dioxide–lignin. The most visible impact is noticeable in a low-frequency range, which is related to the conductivity. Increases in frequency cause decreases in permittivity values in all of the SiO${}_{2}$L-PLA nanocomposites. The higher the silicon-dioxide–lignin content in the polymer matrix, the slower the decrease observed. The temperature effect is also distinctly visible, and is presented in Figure 5, where the real part of the permittivity of the SiO${}_{2}$L-PLA nanocomposites versus the temperature at selected frequencies (0.1 Hz, 10 Hz, 1 kHz, and 1 MHz) is shown.

**Figure 6:**
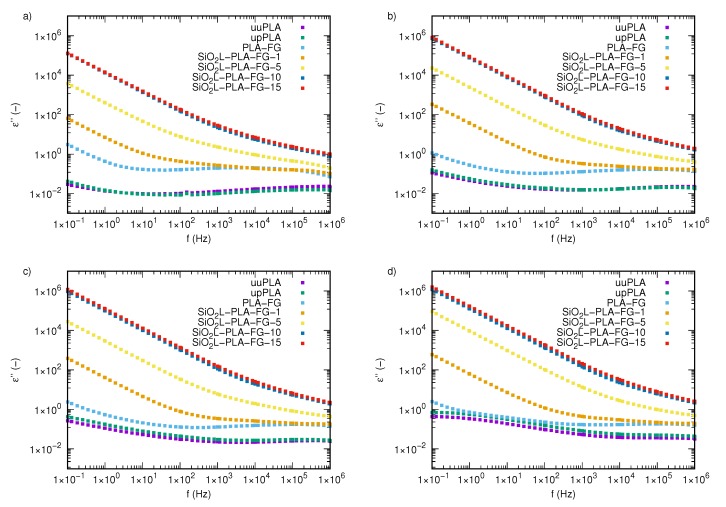
The imaginary part of the complex permittivity of silicon-dioxide–lignin nanocomposites as a function of frequency for (**a**) 293.15 K, (**b**) 323.15 K, (**c**) 328.15 K, and (**d**) 333.15 K.

**Figure A3:**
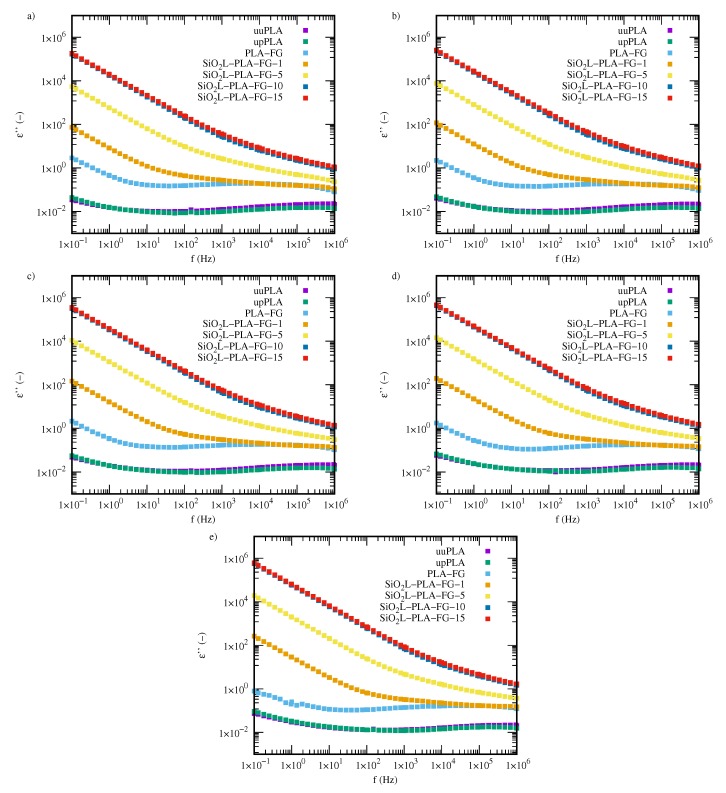
The imaginary part of the complex permittivity of silicon-dioxide–lignin nanocomposites as a function of frequency for (**a**) 298.15 K, (**b**) 303.15 K, (**c**) 308.15 K, (**d**) 313.15 K, and (**e**) 318.15 K.

The imaginary part of the permittivity, $\varepsilon {}^{\prime \prime }$, as a function of frequency in all tested samples, is presented in Figure 6 and Figure A3. In contrast to the real part of the permittivity, the imaginary part, for both processed and unprocessed PLA, is slightly susceptible to the frequency and temperature changes. For temperatures below 323.5 K (Figure 6c), the imaginary permittivity curves of uuPLA and upPAL are very similar. The main difference is visible above 1 kHz, where processed PLA begins to take values slightly lower than uuPLA, which can be observed in Figure 6a and Figure A3. In frequencies below 10 Hz and temperatures between 293.15 and 323.15 K (Figure 6a–c), the behavior of uuPLA and upPLA under an alternating electric field is the same, and a decrease in frequency results in a slight increase in $\varepsilon {}^{\prime \prime }$. Clear differences between uuPLA and upPLA appear when the temperature rises above 328.15 K (Figure 6d), and the imaginary part of the permittivity of processed polylactide is higher than those for uuPLA in the entire frequency window. The temperature effect is also much stronger above this temperature, especially at low frequencies.

As can be seen in Figure 6a,b, adding glycerine to PLA has a large impact on its dielectric properties. There is a clear difference between pure polylactide samples and the mixture of glycerine and PLA over the entire range of tested frequencies. The addition of 20 wt % glycerine to polylactide results in higher values for the imaginary part of the permittivity. First, by increasing frequency, the imaginary part of the permittivity decreases, increases slightly, and then, decreases again at the highest frequency tested–this trend is particularly visible at lower temperatures.

All SiO${}_{2}$L-PLA-FG nanocomposites show that the nanoparticle filler clearly affects the dielectric properties. The higher the mass fraction of the silicon-dioxide–lignin nanoparticles in the nanocomposite matrix, the greater the impact on the imaginary part of the permittivity. Polylactide nanocomposites with 1 wt % SiO${}_{2}$L nanoparticles dispersed in the polymer matrix exhibit a strong decrease in the imaginary part of the permittivity when the frequency increases to 100 Hz. If the frequency is higher than 100 Hz, the effect is much milder.Samples containing 5, 10, and 15 wt% SiO${}_{2}$L nanoparticles show decreasing values in the imaginary part of the permittivity with increasing frequency, and this occurs for almost the entire range of frequencies tested. Interestingly, nanocomposites with 10 and 15 wt% silicon-dioxide–lignin nanoparticles are characterized by highly similar values to those of the imaginary part of the permittivity. For higher frequencies, a slight increase in $\varepsilon {}^{\prime \prime }$ in the SiO${}_{2}$L-PLA-FG-15 nanocomposite is visible.

**Figure 7:**
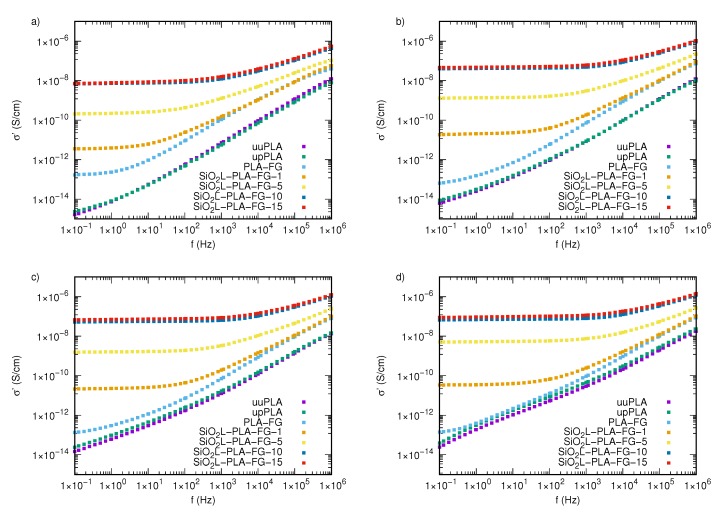
The real part of the complex conductivity of silicon-dioxide–lignin nanocomposites as a function of frequency for (**a**) 293.15 K, (**b**) 323.15 K, (**c**) 328.15 K, and (**d**) 333.15 K.

**Figure A4:**
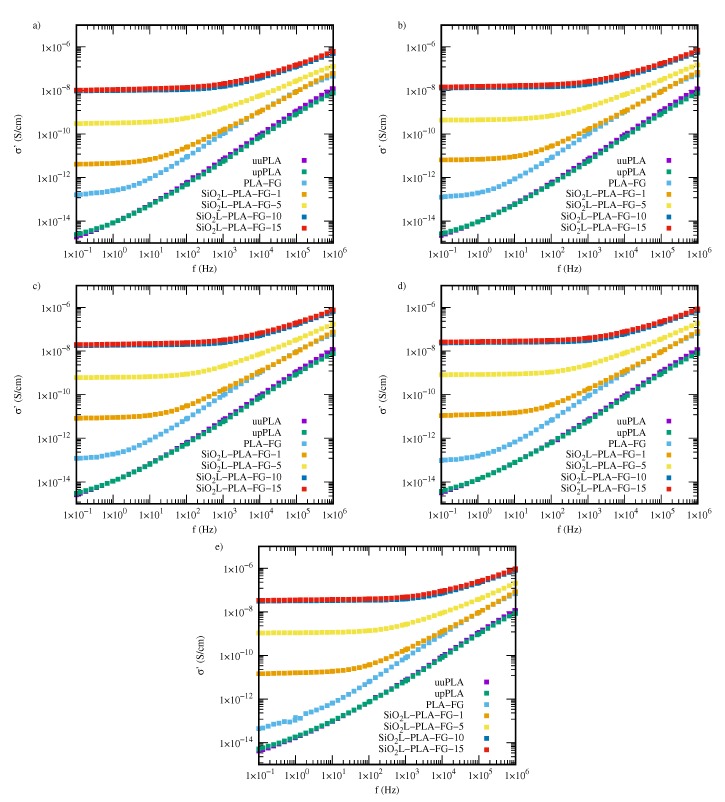
The real part of the complex permittivity of silicon-dioxide–lignin nanocomposites as a function of frequency for (**a**) 298.15 K, (**b**) 303.15 K, (**c**) 308.15 K, (**d**) 313.15 K, and (**e**) 318.15 K.

The real part of the complex electrical conductivity, $\sigma {}^{\prime }$, as a function of frequency for the SiO${}_{2}$L-PLA-FG nanocomposites with various mass fractions of filler at temperatures between 293.15 and 333.15 K, is shown in Figure 7 and Figure A4. As in the case of previously studied electrical properties of these nanocomposites ($\varepsilon {}^{\prime }$, $\varepsilon {}^{\prime \prime }$), in the case of electrical conductivity, there is no significant difference between processed and unprocessed polylactide. The pure polymer matrix, without glycerine or nanoparticles, is characterized by similar behavior under frequency and temperature changes. The difference becomes visible only above the temperature of 328.15 K (Figure 7c), where upPLA reaches higher values of electrical conductivity in the entire tested frequency range.

**Figure 8:**
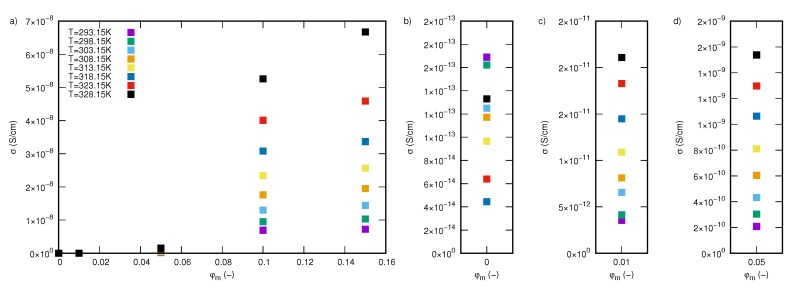
The dependence of the electrical conductivity at 0.1 Hz on the mass fraction of SiO${}_{2}$L nanoparticles: (**a**) in the entire mass fraction range, (**b**) the magnification for 0 mass fraction, (**c**) the magnification for 0.01 mass fraction, (**d**) the magnification for the 0.05 mass fraction.

**Figure 9:**
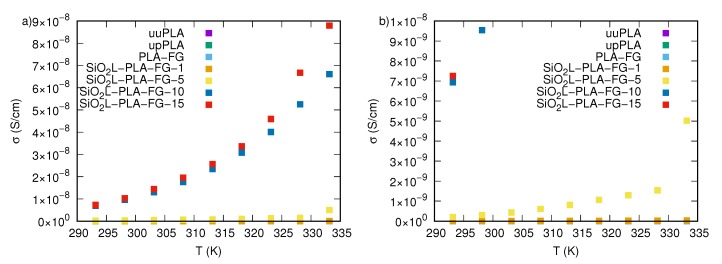
The dependence of the electrical conductivity at 0.1 Hz on the temperature of the unfilled and SiO${}_{2}$L-PLA-FG nanocomposites: (**a**) for the entire electrical conductivity range, (**b**) for the electrical conductivity range between 0 and 10${}^{-8}$ Scm${}^{-1}$.

The experimental data clearly indicate that adding silicon-dioxide–lignin nanoparticles and glycerine to polylactide significantly improves the electrical conductivity of those nanocomposites in the entire tested frequency window. The most visible effect of the SiO${}_{2}$L nanoparticles manifests itself in creating regions where frequency changes do not affect $\sigma {}^{\prime }$ values. These areas are called plateaus, and they are related to direct current (DC) conductivity, $\sigma_{DC}$. The width of such a region is dependent on the amount of silicon-dioxide–lignin nanoparticles in the polymer matrix. The higher the mass fraction of filler, the wider the region that is unaffected by frequency changes. The second effect is a significant increase in the electrical conductivity with an increasing mass fraction of filler, which causes higher electrical conductivity by several orders of magnitude, compared with that of unfilled polylactide, particularly at low frequencies of an external alternating electrical field. The dependence of $\sigma^{\prime }$ at 0.1 Hz on the mass fraction of filler is shown in Figure 8, where it is clearly visible that the most significant enhancement in electrical conductivity occurs in the SiO${}_{2}$L-PLA-FG-10 and SiO${}_{2}$L-PLA-FG-15 nanocomposites in the entire examined temperature window. The electrical conductivity of these nanocomposites is also affected by temperature (Figure 9). This increase causes an increase in values of $\sigma {}^{\prime }$ and affects the width of the plateau. For SiO${}_{2}$L-PLA-FG nanocomposites, an increase in temperature widens the frequency-independent area.

For PLA-FB, an increase in temperature results in higher values of electrical conductivity and the simultaneous disappearance of the plateau. The obtained electrical conductivity spectra meet the universal power law expressed by the following equation [39]: $\sigma \left(\omega \right)=\sigma_{DC}+A\omega^{n}$, where *A* is a numerical factor, *n* is an exponent with a value between 0 and 1, and ω=2πf is the angular frequency. $\sigma_{DC}$ can be obtained as an average from the region that is insensitive to frequency changes. In such a case, where this region is not present, this value can be taken as the value of $\sigma {}^{\prime }$ at the lowest frequency [25]. Reports from other researchers show that nanocomposite-filled nanoparticles are in accordance with the universal power law. Laredo et al. showed this for poly(ε-caprolactone)/polylactide with multi-walled carbon nanotubes [25], Wongtimnoi et al. showed this for polyurethane with carboxyl-functionalized multi-walled carbon nanotubes [40], and other researchers have shown this for other nanocomposites as well [41,42,43].

## 3. Materials and Methods

### 3.1. Materials

Natural and colorless polylactide (PLA) (Proprox, Chwaszczyno, Poland) was supplied as a white 3D printing filament with a diameter of 1.75 mm and was palletized. Hybrid silicon-dioxide–lignin (SiO${}_{2}$L) nanoparticles were produced by mixing an appropriate amount of both ingredients and grinding them to improve homogeneity and reduce the size of the particles. Specific information about the preparation procedure of silicon-dioxide–lignin nanoparticles can be found elsewhere [44,45]. The maleic anhydride grafted polyethylene (F) (Fusabond E226, DuPont, Wilmington, DE, USA) with 0.5 wt % was used as a compatibilizer. To obtain better plasticity and improve the electrical conductivity, 20 wt % anhydrous glycerine, labeled as G (Chempur, Piekary Śląskie, Poland), was used. The specific proportions of ingredients and the labels of prepared samples are summarized in Table 1.

### 3.2. Sample Preparation

The first appropriate amounts of ingredients were weighted with an analytic balance (Pioneer Semi-Micro PX225DM, OHAUS Corporation, Parsippany, NJ, USA) with an accuracy of 0.01 mg and mixed. After that, the composition was melt-blended with a co-rotating twin-screw extruder (HAAKE MiniLab II, Thermo Fisher Scientific, Karlsruche, Germany) at 463.15 K, with a rotation speed of 50 rpm. Samples of unfilled PLA were prepared as well, with the same processing condition. Extruded nanocomposites were palletized and were then injection-molded into bars with 10 × 60 × 1 mm dimensions using a HAAKE MinJet II (Thermo Fisher Scientific, Karlsruche, Germany). The temperature of the cylinder was set to 483.15 K and 333.15 K for the mold. Plasticization time was 2 min, and the injection pressure and time were set, respectively, to 950 bar and 5 s. Additionally, one sample of unfilled PLA was prepared directly by injection molding (without previous extrusion) and was labeled as uuPLA. Using a heated press and cylindrical knife, samples were formed into a disc with a 20 mm diameter.

### 3.3. Characterization Methods

The structural characterization of the SiO${}_{2}$L-PLA-FG nanocomposites was carried out using scanning electron microscope (SEM) Hitachi S-3400N (Hitachi Ltd, Tokyo, Japan). SEM observation was conducted in high- and low-vacuum mode (LV-50 Pa) with a backscattered electron detector (BSE) and a 5 kV accelerating voltage.

Light absorbance of silicon-dioxide–lignin polylactide nanocomposites was measured with a UV-VIS spectrometer Evolution 220 (Thermo Fisher SCIENTIFIC, Waltham, MA, USA). Measurement was conducted in transmittance mode in a range from 190 to 1100 nm with a 2 nm gap and an integration time of 0.05 s. The scanning speed was set to 1200.00 nm/min.

The electrical properties were measured with a broadband spectroscopy device (Concept 80 System, Novocontrol GmbH, Montabaur, Germany). The frequency was changed from 0.1 Hz to 10 MHz in 55 steps using a logarithmic scale. The temperature range was stabilized with a 0.3 K accuracy, and measurements were performed from 293.15 to 333.15 K with a 5 K step. Samples, in the form of a disc with a diameter of 20 mm, were placed between two brass electrodes and disposed in the chamber with temperature stabilization. More details about this setup with a schematic diagram can be found elsewhere [46].

## 4. Conclusions

Nanocomposites based on a blend of polylactide and glycerine with the addition of various mass fractions of silicon-dioxide–lignin (SiO${}_{2}$L) were prepared by melt blending and injection molding. The electrical properties of these nanocomposites were studied with broadband dielectric spectroscopy at a temperature range between 293.15 and 333.15 K. Experimental results clearly show that the combination of silicon-dioxide–lignin nanoparticles and glycerine affects the electrical properties of the nanocomposite matrix with filler at all frequencies and at all temperatures. The real and imaginary parts of the permittivity are greatly affected by the increasing mass fraction of SiO${}_{2}$L filler, particularly at low frequencies. This effect becomes weaker as the frequency increases within the tested temperature range. An increase in silicon-dioxide–lignin content in the nanocomposite matrix also has a substantial impact on the electrical conductivity, and the obtained curves are consistent with the universal power law. Depending on the mass fraction, the electrical conductivity can increase by several orders of magnitude. The most favorable effect of SiO${}_{2}$L nanoparticles was observed at the two highest mass fractions tested (10 and 15 wt %).

## Figures and Tables

**Table 1:** Labels and proportions of ingredients of prepared nanocomposite samples in mass fraction.

Full Name of Ingredients	Label	NP	Fusabond	Glycerine	PLA
unfilled (u) and unprocessed (u) PLA	uuPLA	-	-	-	1
unfilled (u) and processed (p) PLA	upPLA	-	-	-	1
mixture of PLA, Fusabond (F), glycerine (G)	PLA-FG	-	0.005	0.2	0.795
silicon-dioxide–lignin (1 wt %) PLA	SiO${}_{2}$L-PLA-FG-1	0.01	0.005	0.2	0.785
silicon-dioxide–lignin (5 wt %) PLA	SiO${}_{2}$L-PLA-FG-5	0.05	0.005	0.2	0.745
silicon-dioxide–lignin (10 wt %) PLA	SiO${}_{2}$L-PLA-FG-10	0.10	0.005	0.2	0.695
silicon-dioxide–lignin (15 wt %) PLA	SiO${}_{2}$L-PLA-FG-15	0.15	0.005	0.2	0.645
